# Metabolic dysregulation and antibody-mediated rejection after kidney transplantation: interacting mechanisms and emerging clinical strategies

**DOI:** 10.3389/fimmu.2026.1826005

**Published:** 2026-05-21

**Authors:** Qizhen Yang, Shuai Tang, Kejing Zhu, Yulin Niu

**Affiliations:** 1 Organ Transplantation Department, The Affiliated Hospital of Guizhou Medical University, Guiyang, Guizhou, China; 2 Guizhou Medical University, Guiyang, Guizhou, China

**Keywords:** antibody-mediated rejection, dyslipidemia, hypertension, kidney transplantation, metabolic syndrome, post-transplant diabetes mellitus

## Abstract

**Background:**

Kidney transplantation (KT) improves survival in end-stage renal disease, but long-term outcomes are undermined by antibody-mediated rejection (ABMR) and metabolic complications. Recent insights suggest a mechanistic interplay between immune injury and metabolic dysregulation.

**Methods:**

This narrative review synthesizes current literature on the pathogenesis and clinical impact of antibody-mediated rejection (ABMR), the epidemiology of post-transplant metabolic syndrome—including post-transplant diabetes mellitus, dyslipidemia, and hypertension—and their bidirectional relationship. Emerging diagnostic tools and therapeutic strategies are also examined.

**Results:**

ABMR remains a leading cause of late graft failure. Concurrently, 30% ~ 50% of KT recipients develop metabolic syndromes within the first year, driven by immunosuppressive agents and underlying risk factors. Metabolic abnormalities enhance endothelial activation and complement-driven inflammation, aggravating ABMR. Conversely, immunosuppressive intensification to treat ABMR worsens metabolic profiles, forming a vicious cycle. Novel immunologic (e.g., complement inhibitors, anti-CD38 antibodies) and metabolic (e.g., SGLT2 inhibitors, GLP-1 receptor agonists) agents show promise, though data on long-term efficacy remain limited.

**Conclusion:**

Integrated immunometabolic strategies are essential for optimizing graft and patient survival. Future research should focus on personalizing immunosuppression and targeting metabolic health to break the feedback loop linking ABMR and metabolic disease.

## 1 Introduction

Kidney transplantation (KT) provides eligible patients with end-stage kidney disease the best opportunity for long-term, dialysis-free survival at the lowest cost to the health care system (1), but long-term graft outcomes are tempered by both immunological and metabolic challenges (2, 3). According to the 2022 Annual Report of the Organ Procurement and Transplantation Network/Scientific Registry of Transplant Recipients (OPTN/SRTR), among adult kidney transplant recipients, the 5-year graft survival rates for living donor kidney transplantation (LDKT) and deceased donor kidney transplantation (DDKT) were 90.0% and 81.4%, respectively, in the 18–34 age group, declining to 80.8% and 67.8% in the ≥65 age group (4). A comparable trend is seen in the Asia-Pacific region: the Korean Organ Transplantation Registry (KOTRY) reported no further improvement in long-term survival for >6,000 kidney recipients (64.8% with living donors and 35.2% with deceased donors) registered from 2014 to 2019 (5). Chronic graft attrition continues to limit long-term success, necessitating a broader understanding of the factors influencing graft health. Antibody-mediated rejection (ABMR), driven by donor-specific antibodies against the graft, has emerged as a leading cause of late allograft loss despite modern immunosuppressive therapy (6). Concurrently, metabolic complications are highly prevalent among transplant recipients. Studies show that up to 30% ~ 50% of kidney transplant recipients develop metabolic syndrome—including post-transplant diabetes mellitus (PTDM), dyslipidemia, and hypertension—within the first year after transplantation, which collectively increase cardiovascular risk and may adversely impact graft survival (7–9). These complications arise not only from traditional risk factors such as age and obesity, but also from immunosuppressive regimens, especially calcineurin inhibitors and corticosteroids (2, 3). Importantly, increasing evidence suggests that metabolic dysregulation and alloimmune mechanisms are not independent but mechanistically intertwined. Post-transplant metabolic syndrome induces a pro-inflammatory state that is accompanied by oxidative stress, insulin resistance, and endothelial dysfunction and can potentially predispose the graft to immunologic injury (3, 10). As an illustration, hyperglycemia augments advanced glycation end-products and endothelial activation, and dyslipidemia drives complement activation and vascular inflammation—amplifying graft vulnerability to ABMR (11). Management of ABMR, conversely, invariably requires enhanced doses of immunosuppressive therapy, which may exacerbate metabolic imbalance, hence creating a vicious feedback loop. There is growing recognition of this dual-threat paradigm—where metabolic derangement enhances immune-mediated rejection, and immunosuppression further impairs metabolism—demanding integrated management. A recent comprehensive review by Tao et al. elegantly detailed the molecular pathways through which metabolic syndrome reprograms immune cell function across various organ transplants (12). Building upon this mechanistic foundation, the present review focuses specifically on kidney transplantation and the bidirectional clinical interplay between antibody-mediated rejection (ABMR) and post-transplant metabolic syndrome. We aim to provide a clinically oriented synthesis of epidemiology, diagnostic considerations, and evolving management strategies—including novel antidiabetic agents and complement inhibitors—that are particularly relevant to the practicing transplant nephrologist. In this review, we focus on the pathogenesis and clinical significance of ABMR, the spectrum of metabolic derangement after kidney transplant, their mechanistic overlap, and promising strategies to improve patient and graft survival.

## 2 Antibody-mediated rejection: pathogenesis and clinical relevance

ABMR is an antibody-mediated injury of the allograft provoked by donor-specific antibodies (DSA), in the vast majority human leukocyte antigen (HLA) molecules on graft endothelium. DSA binding triggers a cascade of immune events, including inflammation cell influx and activation of the classical pathway of the complement (13). Complement split products (e.g., C4d) deposit in peritubular capillaries as a marker of antibody-mediated kidney transplant injury (14). Downstream effectors such as natural killer (NK) cells play a central role in mediating graft damage by antibody-dependent cellular cytotoxicity in ABMR (11). Complement blockade has become a target of ABMR treatment over the last few years; a Bayesian network meta-analysis in 2024 comparing induction therapies that identified complement inhibition as an effective adjunct in high-risk situations (15).

Clinically, ABMR may arise acutely (days to weeks post-transplant) or as chronic active ABMR months to years post-transplant. Acute and chronic ABMR share a close relationship with graft dysfunction and allograft survival impairment (6, 11). Notably, chronic ABMR, often mediated by post-transplantation *de novo* DSAs, has increasingly been identified in the contemporary era as a leading cause of late allograft failure (6). Histologically, ABMR is characterized by microcirculation changes such as glomerulitis and peritubular capillaritis, often with C4d deposition, and varying degrees of transplant endarteritis and interstitial fibrosis/tubular atrophy in chronic forms (14, 16). These changes reflect antibody-driven graft injury. Notably, the Banff 2022 Kidney Meeting Report introduced important revisions to the diagnostic classification of ABMR, reappraising the diagnostic significance of microvascular inflammation (MVI) and establishing two new diagnostic phenotypes beyond traditional histological criteria: (1) “Probable antibody-mediated rejection (AMR)” —defined as donor-specific antibody (DSA)-positive cases with some histologic features of AMR but below current thresholds for a definitive AMR diagnosis, with negative C4d staining; and (2) “Microvascular inflammation, DSA-negative and C4d-negative” —a phenotype of unclear etiology characterized by microvascular inflammation without evidence of DSA or C4d deposition (17). Furthermore, the Banff 2022 classification incorporates biopsy-based transcript diagnostics as an adjunctive tool for ABMR diagnosis, although further validation is required before routine clinical implementation. These updates facilitate the identification of a broader spectrum of antibody-mediated injury, particularly C4d-negative and DSA-negative microvascular inflammation, thereby improving the diagnostic sensitivity for ABMR. Recent clinical evidence demonstrates that patients diagnosed with MVI (DSA-negative/C4d-negative) or probable AMR according to the 2022 Banff classification have a higher cumulative incidence of ABMR over a median follow-up of 5 years compared with patients without MVI, although their risk of graft loss remains lower than that of patients with active ABMR (18).

Several conditions can predispose candidates to ABMR, including insufficient immunosuppression, HLA incompatibility, and non-HLA antibody presence. For example, endothelial cell antigens or angiotensin II receptor type 1 antibodies have been implicated in refractory rejection situations, which highlights that alloimmune responses can supplant HLA (19). The latest review from Lefaucheur et al. emphasized the role of non-HLA antibodies to promote endothelial activation and prolonged chronic graft damage, i.e., in patients with sustained DSA-negative rejection (20). Despite advances in understanding its pathogenesis, ABMR remains challenging to treat. Treatment of ABMR is generally standard therapy with plasmapheresis and IVIG to remove or neutralize circulating antibodies, and concurrent treatment by immunosuppressive agents in the form of corticosteroids at high doses and B cell- or plasma cell-directed agents (e.g., rituximab, bortezomib) (21). Long-term result remains less than optimal. A randomized controlled trial by Stegall et al. revealed that while Eculizumab reduced histologic damage temporarily in chronic ABMR, there was no improvement in long-term graft function, which indicates the limitations of monotherapy complement inhibition (22). Recent studies have contributed to research on molecular mechanisms of ABMR. For instance, Diebold et al. demonstrated chronic ABMR involves sustained NK cell activation and profibrotic signaling regardless of the classical complement cascade (11). This is supplemented by earlier work by Sorohan et al., whose review addressed complement activation pathways at length and their diagnostic and therapeutic relevance in ABMR (13). At the same time, Zhao et al. discussed immunopathological roles of NK cells in ABMR and described possible NK-targeted therapies (23). Moreover, donor-derived cell-free DNA and transcriptomic profiling have been added as a minimally invasive tool to detect active antibody-mediated damage, possibly guiding earlier intervention (24). Also, a meta-analysis by Abuazzam et al. described the efficacy of many promising future therapies for ABMR—e.g., complement inhibitors, CD38-directed drugs, and immune adsorption—though data on long-term outcomes are limited (6). These studies also mention greater insight into ABMR beyond histology and the need for dynamic immune monitoring. A phase 2 RCT showed that after 6 months of felzartamab, 82% of patients with chronic active ABMR achieved histologic resolution (vs. 20% placebo), with reduced microvascular inflammation and dd-cfDNA levels and acceptable safety. Molecular follow-up of the same trial demonstrated that felzartamab selectively suppresses IFN-γ-inducible and NK cell transcripts, reducing molecular ABMR scores, though some patients relapsed after discontinuation (25, 26). IL-6 pathway blockade is another investigational approach. Clazakizumab showed initial promise in phase 2, but the phase 3 IMAGINE trial was terminated for futility; the phase 3 INTERCEPT trial of tocilizumab is ongoing. A recent case series reported their impact on molecular ABMR scores (27).

## 3 Metabolic dysregulation in kidney transplant recipients

Metabolic health often differs post-transplantation, and many recipients acquire features of metabolic syndrome. Weight gain during the initial post-transplant year is common, and up to 30% of patients fulfill criteria for new-onset metabolic syndrome by this point (9). Post-transplant metabolic syndrome refers to the clustering of conditions such as central obesity, hyperglycemia, dyslipidemia, and hypertension, which can act synergistically to worsen cardiovascular outcomes and possibly graft outcomes. After receiving a kidney transplant, recipients who developed *de novo* metabolic syndrome had a significantly higher risk of graft loss than those who remained metabolically healthy, according to a nationwide cohort study (9). This metabolic derangement is exacerbated by immunosuppressive drugs: corticosteroids promote weight gain and glucose intolerance, calcineurin inhibitors, particularly tacrolimus, suppress insulin secretion and cause hypertension, and mTOR inhibitors cause dyslipidemia (2, 3). Metabolic risk factors are reversible, and cardiovascular disease is the major cause of death in transplant recipients; therefore, diagnosis and management of metabolic dysregulation need to be performed (7, 8). The clinical importance and pathophysiology of the three most important renal transplant metabolic complications—post-transplant hypertension, dyslipidemia, and PTDM—are discussed below.

### 3.1 Post-transplant diabetes mellitus

PTDM, or NODAT, is a frequent complication of KT. Rates of PTDM have differed by population and diagnostic criteria, but approximately 10% ~ 30% of non-diabetic kidney transplant recipients develop diabetes in the first year after transplantation (28, 29). A 2024 systematic review and meta-analysis by Du et al. found that the global pooled prevalence of PTDM was approximately 24.3%, with significant variation across regions and immunosuppressive protocols, highlighting the persistent burden of this complication (30). Both traditional risk factors (older age, obesity, family history of diabetes) and transplantation-specific factors are risk factors for PTDM. Of these, immunosuppressive therapy is most significant: calcineurin inhibitors (notably tacrolimus) are directly diabetogenic via impairment of pancreatic beta-cell function and insulin secretion, and corticosteroids induce peripheral insulin resistance and weight gain (2, 28). mTOR inhibitor drugs can also exacerbate dysglycemia in some cases. Additionally, the transplant operation itself—correction of uremia and high-dose steroid administration in the perioperative period—can unmask a tendency toward hyperglycemia.

PTDM affects both graft and patient outcomes adversely. PTDM has a higher risk of infection, cardiovascular events, and adversely affects the functioning of the kidney graft (29). Ahmed et al. reported that PTDM is significantly associated with higher graft loss and mortality following kidney transplant (10). Rudzki et al. also reported that PTDM adversely affects long-term graft function and causes cardiovascular complications (29). In 2023, an Odler et al. randomized controlled trial demonstrated that the early initiation of basal insulin therapy in high-risk kidney transplant recipients improved short-term glycemic control and health-related quality of life, with implications for the advantages of early active management (31). Mechanistically, chronic hyperglycemia may amplify oxidative stress and inflammatory pathways, which play a potential role in vascular injury within the allograft. Therefore, tight glycemic control is indicated, although it is not yet absolute that evidence supporting a linkage between tighter glucose control and better graft immunologic outcomes is available. Some studies have, however, implied an immunometabolic interface in PTDM: e.g., Lefaucheur et al. underlined that glycation of the endothelium with resultant endothelial activation and microvascular inflammation may lower the threshold for rejection in metabolically unstable recipients (22).

PTDM management is usually the same as type 2 diabetes in non-transplant recipients with suitable adjustments in the transplant environment. Lifestyle modification (diet therapy, weight, and exercise) is the foundation. Pharmacologic management begins with oral hypoglycemic agents such as metformin when renal function is normal or insulin therapy, especially in the early post-transplant period if hyperglycemia is severe. Clinicians can also consider modifying the immunosuppressive regimen to decrease PTDM—e.g., using the lowest feasible dose of tacrolimus, corticosteroid tapering or withdrawal early, or conversion to belatacept in specific cases—although these modifications need to balance rejection risk (32, 33). More novel anti-diabetic drugs, including SGLT2 inhibitors and GLP-1 receptor agonists, have shown potential benefits in the management of PTDM in kidney transplant recipients. Their additional cardiovascular and renal protective effects as well as anti-inflammatory effects may provide additional advantages for the transplant population (3, 33–35). The specific mechanisms, clinical evidence, and safety considerations of these two types of drugs will be elaborated in the subsequent section ‘Metabolic Intervention’.

### 3.2 Dyslipidemia

Dyslipidemia is extremely common after KT, and most recipients develop hypercholesterolemia and/or hypertriglyceridemia post-transplant (4). Both pretransplant lipid disease, diet, weight gain, and immunosuppressive side effects are contributory. Corticosteroids exacerbate hyperlipidemia, and calcineurin inhibitors (especially cyclosporine) can raise LDL cholesterol and worsen lipid clearance. mTOR inhibitors are particularly known to induce hypertriglyceridemia and hypercholesterolemia (3, 7). As a result, many transplant patients meet criteria for dyslipidemia requiring intervention.

Post-transplant dyslipidemia is of severe consequence. Cardiovascular disease is the leading cause of mortality in renal transplant patients, and dyslipidemia is a proven risk factor for atherosclerotic cardiovascular disease in such patients (8). Uncontrolled dyslipidemia may also contribute to graft dysfunction over the long term by promoting renal allograft vasculopathy and glomerulosclerosis, although immunologic injury usually plays a larger role in chronic graft changes. Importantly, managing dyslipidemia in transplant patients has been shown to improve outcomes. A meta-analysis and systematic review in 2024 confirmed again that statin treatment has a big impact on lipid profiles in kidney transplant patients, with evidence for reduced cardiovascular events and all-cause mortality but not graft survival (36). In keeping with the results of recent research by Chmielnicka et al., the authors emphasized the extremely frequent presence of dyslipidemia in transplant recipients and the need for individualized lipid-lowering strategies with consideration of both metabolic profiles and immunosuppressive interactions (37). In addition to statins, other agents such as ezetimibe or PCSK9 inhibitors could be considered in resistant patients to achieve LDL cholesterol goals based on evidence extrapolated from the general population and early experience in transplant recipients (38, 39).

Individualized and strict control of post-transplant dyslipidemia is warranted. Exercise and heart-healthy diets are advised in all transplant recipients (4). In those who need pharmacotherapy, however, clinicians must exercise caution regarding drug–drug interactions between lipid-lowering drugs and immunosuppressants. For instance, calcineurin inhibitors may lead to an increase in serum statin levels, and therefore, statin toxicity can be enhanced. Initiation with low-dose statins and titration, thus, is prudent (7). Regular monitoring of lipid panels and liver enzymes is needed upon initiation of therapy (39). Moreover, novel interventions such as vitamin K supplementation have been explored for vascular health optimization; however, the ViKTORIES randomized controlled trial found no significant improvement in arterial stiffness or coronary calcium scores among kidney transplant recipients receiving vitamin K2 supplementation (40). Lastly, active treatment and diagnosis of dyslipidemia are indicated in kidney transplant recipients as a part of the extensive plan to reduce cardiovascular risk and maintain long-term graft function. In terms of translational advancement, PCSK9 inhibitors such as evolocumab have been found to have very efficacious LDL-lowering activity along with acceptable safety profiles in stable kidney transplant recipients, as per Iannuzzo et al. (38). Early case series during 2024 also support the hypothesis that lipid control may reduce markers of vascular inflammation relevant to graft microcirculation, though future trials are being conducted.

### 3.3 Post-transplant hypertension

Hypertension is highly prevalent among kidney transplant recipients, affecting over 70% of patients within the first post-transplant year (41). Its pathogenesis is multifactorial, encompassing the persistence of pre-existing hypertension, calcineurin inhibitor-induced vasoconstriction and sodium retention, corticosteroid-related fluid retention, and activation of the renin-angiotensin-aldosterone system secondary to graft injury (42, 43). Uncontrolled hypertension is an independent risk factor for both cardiovascular events and graft loss (44, 45).

Lifestyle modification forms the foundation of management. Pharmacologically, RAAS blockade with either an ACE inhibitor or an angiotensin receptor blocker is the preferred first-line therapy, given its combined antihypertensive, antiproteinuric, and renoprotective effects; calcium channel blockers are also commonly employed due to their efficacy in counteracting calcineurin inhibitor-mediated vasoconstriction (7, 42, 46). Most patients require combination therapy to achieve target blood pressure. For resistant hypertension, optimization of the immunosuppressive regimen—such as reducing the dose of tacrolimus or corticosteroids—should be considered provided that the rejection risk remains acceptably low (41, 42). Ambulatory blood pressure monitoring facilitates the detection of masked and nocturnal hypertension, thereby refining risk stratification (46, 47).

There is some debate about the optimal blood pressure targets in transplant recipients, but current practice generally aligns with guidelines for the general CKD population (e.g. goal <130/80 mmHg in most cases) (41, 43, 48). Ambulatory blood pressure monitoring may be useful to detect nocturnal hypertension or masked hypertension in this population, improving risk stratification (46). Ultimately, tight blood pressure control is a key component of post-transplant care to improve patient and graft outcomes. Recent studies have emphasized the role of nocturnal hypertension and blood pressure variability in predicting allograft injury. Kim et al. found that ambulatory blood pressure monitoring (ABPM) identified masked hypertension in over 30% of stable recipients, and uncontrolled nocturnal BP was independently associated with higher graft fibrosis scores (44). In summary, hypertension is a common and significant risk factor for adverse outcomes in kidney transplant recipients. Accurate monitoring using ABPM and individualized treatment strategies are essential for improving patient and graft survival (49).

## 4 Mechanistic interactions between metabolic dysregulation and antibody-mediated rejection

**Figure 1:**
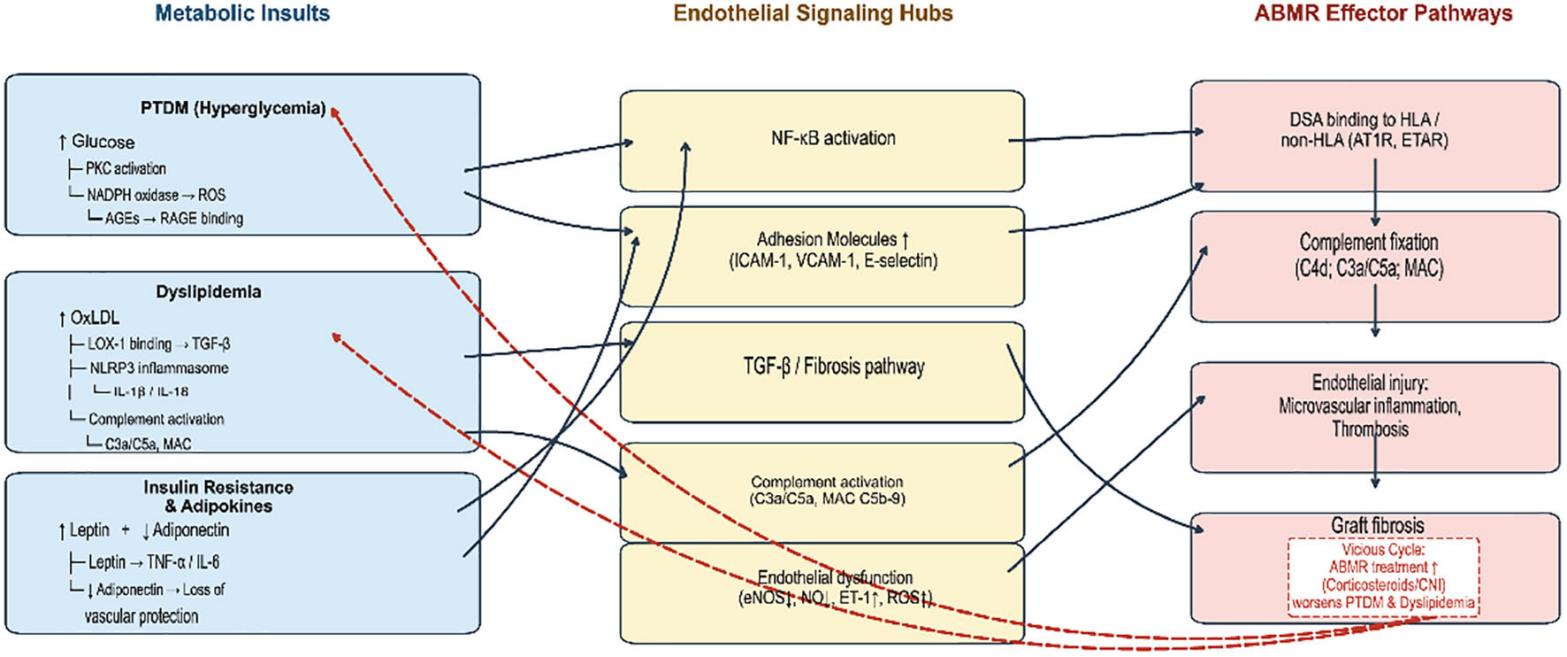
Molecular crosstalk between post-transplant metabolic syndrome and ABMR, antibody-mediated rejection in kidney transplantation. Metabolic derangements—hyperglycemia (PTDM), dyslipidemia, and adipokine imbalance (↑leptin, ↓adiponectin)—converge on the graft endothelium to activate shared signaling hubs: NF-κB-driven adhesion molecule expression (ICAM-1, VCAM-1, E-selectin), LOX-1/TGF-β-mediated fibrotic pathways, ROS/AGE/RAGE-induced oxidative stress, and complement activation (C3a/C5a, MAC C5b-9). Beyond the pathways depicted, AGEs also form irreversible crosslinks with extracellular matrix proteins and deposit within basement membranes, sustaining local immune activation. Oxidative stress further sensitizes the endothelium to DSA by downregulating complement regulatory proteins (CD55, CD59) and upregulating NKG2D ligands (MICA/MICB). Together, these mechanisms constitute the “first hit” of the two-hit hypothesis, priming the graft for amplified injury upon DSA binding (the “second hit”). AGE, advanced glycation end-product; DSA, donor-specific antibody; LOX-1, lectin-like oxidized LDL receptor-1; MAC, membrane attack complex; PKC, protein kinase C; PTDM, post-transplant diabetes mellitus; RAGE, receptor for AGE; ROS, reactive oxygen species.

There is also cumulative evidence that metabolic disease and alloimmune injury in kidney transplant patients are not entirely independent processes. Instead, they are likely to influence one another in complex mechanisms, interweaving at multiple molecular levels. For example, the development of post-transplant metabolic syndrome has been linked with worse transplant outcomes, including increased risk of late allograft failure (9, 50). Those patients developing features of metabolic syndrome (such as PTDM or obesity with dyslipidemia and hypertension) have a pro-inflammatory milieu with insulin resistance, adipokine imbalance, and oxidative stress (3). This environment can potentially lower the threshold for immune-mediated graft injury. The molecular pathways linking these metabolic perturbations to ABMR pathogenesis are depicted in **Figure 1**.

Chronic hyperglycemia, for instance, can lead to advanced glycation end-products formation and endothelial dysfunction that can exacerbate microvascular injury in the setting of ABMR (11). Chronic hyperglycemia in PTDM drives endothelial dysfunction through several convergent signaling cascades. Elevated glucose flux activates protein kinase C (PKC), which in turn stimulates NADPH oxidase to generate excessive reactive oxygen species (ROS). ROS promote the non-enzymatic formation of advanced glycation end-products (AGEs), which engage their receptor RAGE on endothelial cells, activating the NF-κB pathway and upregulating adhesion molecules including ICAM-1, VCAM-1, and E-selectin. These adhesion molecules facilitate leukocyte tethering and transendothelial migration, effectively “priming” the graft endothelium for subsequent immune-mediated attack. AGE accumulation has also been associated with increased complement susceptibility. A recent comprehensive review of endothelial dysfunction in kidney transplantation highlights NADPH oxidase/ROS, AGE/RAGE, and adhesion molecules (ICAM-1/VCAM-1/E-selectin) as key biomarkers and mechanistic drivers in this population (51).

In the same way, dyslipidemia and resultant endothelial deposition of oxidized lipids can also foster complement activation and macrophage recruitment within the graft vasculature, exacerbating any concurrent antibody-mediated injury (13). Post-transplant dyslipidemia, exacerbated by calcineurin inhibitors and mTOR inhibitors, elevates circulating oxidized low-density lipoprotein (OxLDL). OxLDL binds to the lectin-like oxidized LDL receptor-1 (LOX-1) on graft endothelial cells, activating TGF-β signaling and promoting early interstitial fibrosis. In a porcine renal auto-transplantation model, hyperlipidemic diet-induced increases in plasma OxLDL correlated with proteinuria, graft fibrosis, and activation of renal TGF-β signaling, implicating LOX-1 as a potential therapeutic target (52). OxLDL also functions as a damage-associated molecular pattern (DAMP), activating the NLRP3 inflammasome and amplifying IL-1β/IL-18-driven vascular inflammation. Furthermore, dyslipidemia enhances complement-mediated injury within the graft microvasculature. Modified lipoproteins deposited in the subendothelial space activate the classical and alternative complement pathways, generating anaphylatoxins C3a and C5a—potent chemotactic agents that recruit neutrophils and macrophages—and the membrane attack complex (MAC, C5b-9), which directly lyses endothelial cells. A 2025 review on complement activation in kidney transplantation underscores that complement-mediated endothelial injury is central to both ischemia-reperfusion injury and antibody-mediated rejection (53). Notably, emerging evidence indicates that complement-independent mechanisms may be more prominent in late (>6 months) versus early ABMR, suggesting temporal heterogeneity in the molecular pathogenesis of ABMR (54).

Insulin resistance and imbalance of fat factors will further amplify endothelial inflammation. Insulin resistance, driven by corticosteroids and calcineurin inhibitors, is accompanied by profound alterations in the adipokine profile. Elevated leptin levels, commonly observed in kidney transplant recipients with PTDM, correlate with parameters of endothelial dysfunction including reduced nitric oxide bioavailability and increased high-sensitivity C-reactive protein. High leptin signaling promotes endothelial expression of adhesion molecules and pro-inflammatory cytokines, including TNF-α and IL-6. Conversely, the protective adipokine adiponectin—which enhances insulin sensitivity and exerts anti-inflammatory effects—is characteristically reduced in post-transplant metabolic syndrome. Low adiponectin levels are independently associated with insulin resistance and increased PTDM risk (55) This imbalance creates a self-perpetuating cycle: leptin-driven inflammation exacerbates endothelial dysfunction, while adiponectin deficiency removes a critical brake on vascular inflammation, rendering the graft endothelium more susceptible to DSA-triggered injury.

Clinical data offers a foundation for a convergence of metabolic health and graft immunologic outcomes. *De novo* metabolic syndrome after transplant was associated with a higher risk of 5-year graft failure in a large cohort (9). In another study, renal transplant recipients who had pre-transplant diabetes or developed PTDM had poorer graft survival outcomes compared to those who were not diabetic (10). While immunological mismatches and non-adherence remain primary drivers of rejection, these findings indicate that metabolic dysregulation can aggravate graft injury. It is conceivable that suboptimal metabolic control acts as a “second hit” on the graft, accelerating damage in the presence of alloimmune aggression.

Conversely, the relationship is bi-directional: severe graft injury from rejection can worsen metabolic parameters (2, 3). High-dose corticosteroids used to treat rejection episodes can precipitate hyperglycemia and fluid retention, and a failing graft (with reduced renal function) can itself cause hypertension and dyslipidemia to worsen, creating a vicious cycle (2, 7, 10, 41). In addition, efforts to reduce immunologic risk through augmentation of immunosuppression will come at the cost of enhanced metabolic side effects (2, 56). For example, preservation of the more elevated tacrolimus levels is protective against rejection but subjects the patient to PTDM, whereas prolonged use of steroids is protective against rejection at the cost of weight gain, diabetes, and hypertension (2, 56). These metabolic insults converge on the same endothelial substrate targeted by donor-specific antibodies (DSA). In ABMR, DSA binding to HLA or non-HLA antigens (such as angiotensin II type 1 receptor and endothelin A receptor) on graft endothelial cells triggers complement fixation with generation of anaphylatoxins (C3a/C5a) and assembly of the MAC (C5b-9). This antibody binding also directly activates endothelial cells, increasing expression of HLA, non-HLA antigens, and adhesion molecules (ICAM-1/VCAM-1), resulting in microvascular inflammation, thrombosis, and apoptosis (19). A pre-existing metabolically inflamed endothelium—already displaying upregulated adhesion molecules (ICAM-1/VCAM-1/E-selectin), activated NF-κB, and impaired nitric oxide bioavailability—presents a primed target for DSA. The superimposed immune injury from ABMR triggers additional complement activation and inflammatory cell recruitment, further damaging the endothelium. Moreover, the management of ABMR invariably requires intensified immunosuppression (high-dose corticosteroids, augmented CNI dosing), which directly worsens hyperglycemia, dyslipidemia, and hypertension, thereby reinforcing the metabolic-endothelial injury axis.

Beyond activating endothelial NF-κB signaling via RAGE, AGEs exert broader local modulatory effects within the graft microenvironment. AGEs form irreversible crosslinks with extracellular matrix proteins such as collagen and laminin, increasing vascular stiffness and altering endothelial mechanosensation, which further amplifies endothelial responsiveness to inflammatory stimuli (51). Serum AGEs levels in renal transplant recipients correlate with abnormal α-SMA and OPN expression in arterial walls and accelerated arteriosclerosis; AGEs induce vascular smooth muscle cell-to-osteoblast trans-differentiation through the AGE/RAGE/ILK pathway, thereby accelerating post-transplant arteriosclerosis (57). AGE formation is associated with various alloantigen-unrelated risk factors including recipient age, diabetes, proteinuria, hypertension, and hyperlipidemia, and *in vitro* studies have confirmed that AGEs induce the expression of multiple mediators linked to chronic renal transplant dysfunction (58). This AGE-mediated matrix remodeling and immune cell pre-activation collectively constitute a core mechanism of the “first hit” in the “two-hit” hypothesis. Oxidative stress not only upregulates adhesion molecule expression via NF-κB activation but also directly enhances endothelial vulnerability to DSA-mediated injury through multiple pathways. First, ROS downregulate the expression of complement regulatory proteins (e.g., CD55, CD59) on the endothelial surface—CD55 and CD59 act synergistically to inhibit complement-mediated renal ischemia-reperfusion injury, and abrogation of their function leads to MAC-induced microvascular injury and dysfunction (59). Second, oxidative stress induces endothelial autophagy and the unfolded protein response, increasing the expression of stress-induced ligands such as MHC class I chain-related A/B (MICA/MICB), which are recognized by NKG2D receptors on NK cells, thereby amplifying antibody-dependent cellular cytotoxicity. In antibody-mediated rejection, DSA binding to endothelial cells activates the complement cascade via the classical pathway, generating C3a/C5a and MAC, resulting in endothelial injury and microvascular inflammation (60). Kidney transplant recipients exhibit significantly elevated oxidative stress levels, and the presence of anti-graft antibodies correlates with higher oxidative scores (61). Third, ROS may alter the conformational epitopes of HLA molecules through oxidative modification, potentially affecting the affinity and specificity of DSA binding. Collectively, the molecular pathways described above constitute the framework of the “two-hit” hypothesis of kidney allograft injury: the first hit comprises chronic metabolic stress driven by post-transplant metabolic syndrome (hyperglycemia → AGE/RAGE/oxidative stress, dyslipidemia → OxLDL/LOX-1, adipokine imbalance → low-grade inflammation), which, although insufficient to independently trigger acute rejection, persistently “primes” the graft endothelium, rendering it activated, pro-inflammatory, and complement-susceptible. The second hit is triggered by *de novo* or pre-existing DSA binding; against the backdrop of a primed endothelium, the complement fixation and NK cell activation elicited by DSA are markedly amplified, resulting in an earlier onset and more severe ABMR phenotype. The “two-hit” hypothesis, originally proposed in liver transplantation, posits that a coexistent insult upregulates HLA class II target antigens on the microvascular endothelium, explaining why suboptimal donors with lower sensitization levels may still develop acute AMR and why patients with chronic complications may be more susceptible to chronic AMR (62). This hypothesis is equally applicable to kidney transplantation, explaining why, among patients with comparable DSA levels and similar immunosuppressive regimens, those with poor metabolic control often exhibit earlier graft loss and more severe histologic ABMR injury.

In summary, the metabolic-inflammatory cascade—hyperglycemia → PKC/ROS/AGE/RAGE → NF-κB → adhesion molecules; dyslipidemia → OxLDL/LOX-1 → TGF-β/fibrosis; complement → C5a/MAC → endothelial lysis; and adipokine imbalance → leptin ↑/adiponectin ↓ → TNF-α/IL-6 → vascular inflammation—primes the allograft endothelium for ABMR, while ABMR-directed immunosuppression perpetuates the metabolic derangements that initiated this cascade. This vicious cycle is schematically illustrated in **Figure 1**.

Emerging evidence indicates that the gut microbiome and its metabolites play a critical role in post-transplant immune regulation and alloimmune injury. Kidney transplant recipients exhibit marked gut dysbiosis—a multicenter prospective study involving 217 KT recipients demonstrated that microbial diversity and short-chain fatty acid (SCFA)-producing taxa decline prior to acute rejection, with functional analysis and quantitative PCR confirming decreased production potential of propionate and butyrate (63). A multiomics integration study further revealed that the core microbial species in the rejection group were Escherichia coli and Ruminococcus gnavus, whereas Faecalibacterium prausnitzii and Bacteroides ovatus predominated in the dysfunction group, with specific differential species closely linked to host immunological rejection (64) Additionally, a cross-sectional study confirmed that SCFA-producing genera (Faecalibacterium, Roseburia) are altered in KT recipients compared with healthy controls (65).

Furthermore, a prospective study of 97 KT recipients found that fecal concentrations of propionate and lactate were significantly reduced in patients with acute rejection compared with those without rejection, and lipopolysaccharide biosynthesis pathways were notably enriched in the rejection group (66). These processes act synergistically with the endothelial injury driven by hyperglycemia and dyslipidemia, collectively “priming” the graft endothelium and lowering the threshold for antibody-mediated rejection. Moreover, the gut microbiota participates in bile acid metabolism via bile salt hydrolase activity, converting primary bile acids into secondary bile acids that modulate immune cell function through FXR and TGR5 receptors, suppressing Th17 differentiation while promoting Treg expansion and thereby influencing graft immune tolerance (67).

Microbiome-based interventions are actively being explored. A retrospective analysis demonstrated that fecal microbiota transplantation effectively alleviated severe diarrhea and recurrent urinary tract infections in KT recipients secondary to immunosuppressant exposure, with significantly increased relative abundance of beneficial genera such as Faecalibacterium and Roseburia post-FMT (68). Additionally, the ProKiT randomized controlled trial (NCT06825117) is currently evaluating whether probiotic supplementation (OMNi-BiOTiC^®^ 41167) reduces the risk of graft rejection in KT recipients, with primary outcomes including acute rejection incidence, changes in gut microbiota composition, and modification of immunosuppressive regimens. These emerging strategies offer novel opportunities for modulating the post-transplant metabolic–immune axis, though further prospective studies are required to establish their long-term clinical benefit.

Recognition of these interactions has prompted integrated approaches to patient care. Some transplant centers now incorporate early screening and management of metabolic syndrome as part of post-transplant protocols, on the premise that improving metabolic health may improve immunologic outcomes in the long run (3, 56). Interest remains as to whether interventions like weight loss, exercise programs, or use of metabolically favorable immunosuppressants (e.g., the CNI-sparing agent belatacept) could reduce incidence or severity of rejection, although evidence is unsatisfactory (56). However, more holistic consideration of the transplant recipient—understanding that cardiovascular-metabolic well-being and immune-mediated graft well-being are intertwined—is more broadly encouraged (3). Future research will need to reveal the underlying immunometabolic mechanisms connecting these disciplines and to determine if a vigorous control of metabolic risk factors is related to improved graft survival.

**Figure 2:**
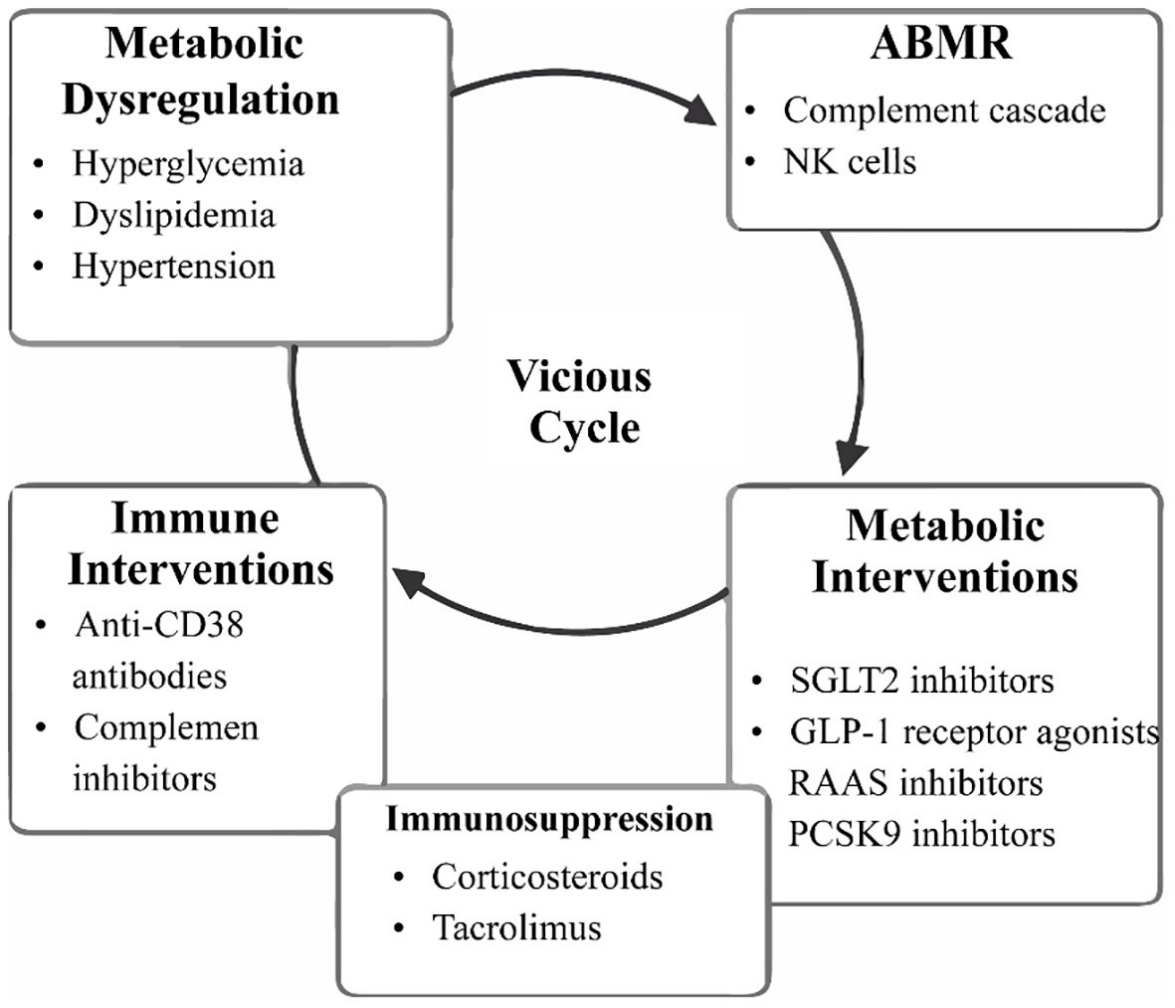
Schematic overview of the bidirectional pathophysiologic loop between metabolic dysregulation and ABMR, antibody-mediated rejection in kidney transplant recipients. Post-transplant metabolic syndrome induces endothelial activation and vascular inflammation, thereby enhancing alloimmune injury through complement activation and NK, natural killer cell cytotoxicity. The intensified immunosuppression required to manage ABMRfurther exacerbates metabolic derangements, perpetuating a vicious cycle. Emerging immunologic therapies and metabolic interventions aim to disrupt this pathogenic loop and improve graft survival. The detailed molecular pathways underlying this crosstalk are illustrated in **Figure 1**.

The interplay between ABMR and metabolic dysregulation is increasingly recognized as a pathophysiologic self-perpetuating cycle. Metabolic derangements—hyperglycemia, dyslipidemia, and hypertension—induce endothelial dysfunction and inflammatory responses within the vasculature and sensitize the graft to alloimmune injury. Treatment of ABMR with augmented immunosuppression deteriorates metabolic profiles and augments systemic risk. **Figure 2** depicts the mechanistic overview of this vicious cycle as well as both immunologic and metabolic therapeutic strategies for interrupting this interaction towards the goal of graft maintenance and patient survival.

## 5 Emerging clinical strategies and interventions

Metabolic risks and antibody-mediated damage in renal transplantation are interdependent aspects and must be optimized for both avenues towards better long-term outcome. Different innovative approaches are being explored:

### 5.1 Advances in ABMR therapy

Conventional methods of ABMR (plasmapheresis, IVIG, rituximab) are limited, particularly in chronic active ABMR (69). New therapeutics against the major pathways of ABMR have been developed in recent years. For example, inhibition of the complement has also emerged as a promising one in challenging cases—eculizumab (monoclonal antibody against C5) has been utilized to prevent and treat acute ABMR in highly sensitized recipients to prevent damage by blocking the complement pathway (13). Another approach is elimination of pathogenic antibodies: the enzyme imlifidase, which cleaves IgG, has been approved in some regions for desensitization and is under investigation for treating refractory ABMR by rapidly reducing DSA levels. Plasma cell-targeted agents, such as the anti-CD38 monoclonal antibody felzartamab, may also help eliminate the source of antibody production. A phase 2 randomized, double-blind, placebo-controlled trial involving 22 patients who developed ABMR at least 180 days post-transplantation demonstrated that felzartamab has an acceptable safety and tolerability profile, with safety and side-effect spectrum as the primary endpoint. Regarding key secondary endpoints, at 24 weeks, 82% (9/11) of patients in the felzartamab group achieved morphologic ABMR resolution compared with 20% (2/10) in the placebo group (risk ratio 0.23; 95% confidence interval [CI] 0.06–0.83); the median microvascular inflammation score was 0 in the felzartamab group versus 2.5 in the placebo group (mean difference −1.95; 95% CI −2.97 to −0.92); and both the molecular ABMR score (0.17 vs. 0.77) and donor-derived cell-free DNA level (0.31% vs. 0.82%) were significantly reduced. Notably, 67% of patients who achieved resolution maintained morphologic regression at 52 weeks without additional therapy, although some patients exhibited molecular recurrence (25). These findings suggest potential therapeutic value for CD38-targeted therapy in chronic active ABMR, though optimal treatment duration and combination strategies require further investigation. Meanwhile, targeting pro-inflammatory cytokines and cells involved in ABMR is being explored—for example, interleukin-6 blockade (tocilizumab) was reported to be effective in chronic active ABMR in early reports, and strategies to deplete or inhibit NK cells (which can effect antibody-dependent damage) are being explored (11, 23). Last but not least, new diagnostics such as donor-derived cell-free DNA analysis and gene expression profiling from blood or urine make possible earlier detection of rejection with scope for early intervention before irreversible damage (6, 24). These new therapies and technologies have the ability to redefine ABMR management outside the traditional plasmapheresis/IVIG paradigm.

Newer agents—including complement inhibitors, anti-plasma cell therapies, and targeted immunomodulators—are being actively explored to improve outcomes in patients with persistent or late-onset ABMR (6, 11, 70). These therapies offer greater precision in modulating pathogenic pathways, though long-term efficacy data are still emerging. At the same time, there is growing interest in combining metabolic control with immune modulation, as accumulating evidence suggests that post-transplant metabolic dysfunction (e.g., diabetes, dyslipidemia, hypertension) can amplify alloimmune injury (3, 33, 71). Novel glucose-lowering drugs such as SGLT2 inhibitors and GLP-1 receptor agonists exhibit anti-inflammatory and renoprotective properties, though their use in transplant populations remains off-label and requires careful monitoring (71). Lipid management with statins and possibly PCSK9 inhibitors, along with Renin–Angiotensin–Aldosterone System (RAAS) blockade for hypertension and proteinuria, may provide both cardiovascular and immunologic benefits (36, 38, 72). The integration of metabolic interventions into ABMR management represents a paradigm shift toward holistic graft preservation. **Table 1** summarizes the key therapeutic strategies currently used or under investigation for the management of ABMR and associated metabolic derangements. This overview includes traditional and emerging immunologic interventions, as well as pharmacologic agents targeting glycemic, lipid, and blood pressure control.

**Table 1:** Summary of therapeutic interventions targeting ABMR, antibody-mediated rejection and metabolic dysregulation after KT, kidney transplantation.

Category	Intervention	Mechanism/target	Key clinical outcomes in KT recipients	Reference
Conventional ABMR	Plasmapheresis + IVIG	Antibody removal/ neutralization	Mainstay for acute ABMR; improves histology	(69)
Conventional ABMR	Rituximab/Bortezomib	B-cell/plasma cell depletion	Mixed results in chronic ABMR	(21)
Conventional ABMR	Eculizumab	Terminal complement inhibition (C5)	RCT: improved histology but no sustained GFR benefit; did not prevent progression to transplant glomerulopathy	(13)
Emerging ABMR	Anti-CD38 (Felzartamab)	Plasma cell targeting	Phase 2 RCT (N = 22): 82% (9/11) achieved morphologic ABMR resolution at 24 weeks vs 20% (2/10) placebo (RR 0.23; 95% CI 0.06–0.83); median MVI score 0 vs 2.5 (mean difference −1.95); 67% maintained resolution at 52 weeks; acceptable safety with mild/moderate infusion reactions	(25, 70)
Emerging ABMR	Cell-free DNA monitoring	Early biomarker for allograft injury	Detects rejection before histologic injury	(6, 21, 24)
Metabolic Interventions	SGLT2 inhibitors	Glycemic control, natriuresis, cardiorenal protection	Efficacy: RCT (EFFiCIS, N = 52): dapagliflozin 10 mg reduced MAP by 3.9 mmHg at week 1, decreased GFR by 4.2 mL/min/1.73m², improved arterial stiffness by 3.5%, increased glucosuria without increased infection risk. Safety concerns: UTI rate ~10% with 5.4% discontinuation for recurrent UTI; initial eGFR dip may cause clinical concern; KTRs excluded from pivotal trials; KDIGO recommendations do not extend to KTRs	(34, 71, 73–77)
Metabolic Interventions	GLP-1 receptor agonists	Glycemic control, weight loss, cardiovascular benefits	Efficacy: Cohort (N = 141, mean 2.4 yrs): weight loss −3.38 kg, BMI −1.28 kg/m² (p<0.001); HbA1c −0.50% in T2DM subgroup (p<0.01); stable eGFR; no acute rejection events. Safety concerns: GI side effects common; may impair oral immunosuppressant absorption, necessitating drug level monitoring; evidence limited by small sample size and short follow-up	(71, 78, 79)
Metabolic Interventions	PCSK9 inhibitors	LDL-C reduction via PCSK9 inhibition	Efficacy: Network meta-analysis (16 RCTs, N = 79,615): evolocumab ranked highest for LDL-C reduction (SUCRA 67.2%) and CVE reduction (69.5%); no increased rejection/infection risk [new citation 4]. Evidence limitations: Data in KTRs limited to case reports/small series; KTRs excluded from major PCSK9i RCTs	(38, 80–82)
Metabolic Interventions	RAAS blockade	Blood pressure and proteinuria reduction	Used in KT for BP and proteinuria control; may offer antifibrotic benefit	

ABMR, antibody-mediated rejection; BMI, body mass index; BP, blood pressure; CI, confidence interval; CVE, cardiovascular event; GFR, glomerular filtration rate; GLP-1 RA, glucagon-like peptide-1 receptor agonist; IVIG, intravenous immunoglobulin; KTR, kidney transplant recipient; KT, kidney transplantation; LDL-C, low-density lipoprotein cholesterol; MAP, mean arterial pressure; MVI, microvascular inflammation; PCSK9i, proprotein convertase subtilisin/kexin type 9 inhibitor; RCT, randomized controlled trial; RR, risk ratio; SGLT2i, sodium-glucose cotransporter-2 inhibitor; SUCRA, surface under the cumulative ranking curve; T2DM, type 2 diabetes mellitus; UTI, urinary tract infection.

### 5.2 Metabolic interventions

On the metabolic front, new interventions seek to reduce risk factors without augmenting immunologic risk. In the context of PTDM, new glucose-lowering agents are being added to post-transplant therapy. SGLT2 inhibitors and GLP-1 receptor agonists, originally developed for type 2 diabetes, confer cardiovascular and renal protection in the general population and are now being tested in transplant recipients with PTDM or metabolic syndrome (3, 33).

#### 5.2.1 Mechanisms of action

SGLT2 inhibitors act by inhibiting sodium-glucose cotransporter 2 in the proximal renal tubule, thereby blocking the reabsorption of glucose and sodium. This produces osmotic diuresis and natriuresis, lowers blood pressure and intraglomerular pressure, and consequently attenuates glomerular hyperfiltration injury. Beyond these hemodynamic effects, SGLT2 inhibitors directly mitigate intra-graft inflammatory responses by improving renal cortical oxygenation, reducing mitochondrial reactive oxygen species production, and inhibiting NLRP3 inflammasome assembly (83). These effects complementarily inhibit the “AGE/RAGE→NF-κB→adhesion molecule” pathway depicted in **Figure 1** of this review, and may theoretically attenuate the metabolic stress-induced “priming” of the graft endothelium.

The anti-inflammatory and anti-fibrotic effects of GLP-1 receptor agonists are achieved through multiple pathways. The GLP-1 receptor is widely expressed on endothelial cells, vascular smooth muscle cells, and immune cells, including T lymphocytes and macrophages. Liraglutide has been shown to inhibit TNF-α-induced NF-κB activation in endothelial cells via the GLP-1R/PKA/cAMP axis, thereby downregulating the expression of VCAM-1 and E-selectin and reducing monocyte adhesion (84). Furthermore, GLP-1 receptor agonists can reduce circulating levels of oxidized low-density lipoprotein and decrease complement C3 deposition in glomeruli and peritubular capillaries, suggesting that they may alleviate complement-mediated injury and vascular inflammation at multiple nodes in ABMR (84).

Taken together, SGLT2 inhibitors and GLP-1 receptor agonists, through independent yet synergistic hemodynamic, metabolic, and anti-inflammatory mechanisms, directly or indirectly modulate multiple molecular nodes—including the NLRP3/NF-κB/adhesion molecule/complement pathways—within the “metabolism-endothelium-ABMR” cascade described in this review.

#### 5.2.2 Clinical evidence

In kidney transplant recipients, the quality of clinical evidence for SGLT2 inhibitors is currently relatively high. The randomized, double-blind, placebo-controlled EFFiCIS trial (n=52) demonstrated that dapagliflozin 10 mg daily for 12 weeks reduced mean arterial pressure by 3.9 mmHg (95% CI −7.5 to −0.2) at week 1, decreased the carotid augmentation index by 3.5% (indicative of improved arterial stiffness), and significantly increased urinary glucose excretion, without an increase in urinary tract or genital infections (34). The acute decline in estimated glomerular filtration rate (eGFR) induced by SGLT2 inhibitors (approximately 3–5 mL/min/1.73 m²) represents a hemodynamically mediated and reversible change, resulting from afferent arteriolar vasoconstriction and reduced intraglomerular pressure, which typically stabilizes or partially recovers with continued treatment (76). However, in the transplant setting, where renal functional reserve is limited, this “pseudo-deterioration” may prompt unnecessary dose reduction or discontinuation, thereby forfeiting long-term cardiorenal protective benefits. Two systematic reviews and meta-analyses have further shown that SGLT2 inhibitors reduce cardiovascular and all-cause mortality in transplant recipients, while also improving HbA1c and body weight (35, 75). Nevertheless, no randomized controlled trials to date have included ABMR-related parameters—such as microvascular inflammation scores, donor-derived cell-free DNA (dd-cfDNA) levels, or *de novo* DSA incidence—as primary or key secondary endpoints (79).

The clinical evidence for GLP-1 receptor agonists is predominantly derived from retrospective studies. A real-world cohort of 141 kidney transplant recipients showed that, over a mean treatment duration of 2.4 years, body weight decreased by 3.38 kg and BMI by 1.28 kg/m² (both p<0.001), with concurrent improvements in total cholesterol and LDL-cholesterol and a significant reduction in systolic blood pressure; HbA1c declined by 0.50% in the subgroup with pre-existing type 2 diabetes (p<0.01), whereas fasting glucose increased slightly in the PTDM subgroup. Overall safety was favorable, with a urinary tract infection rate of 18% (mostly asymptomatic or mild), and no acute rejection, severe hypoglycemia, or treatment-related deaths were reported (78). Available data indicate that GLP-1 receptor agonists are generally well tolerated in this population, with no definitive evidence of an increased risk of graft rejection, acute pancreatitis, or drug-drug interactions with immunosuppressive agents (79). However, gastrointestinal adverse effects—including nausea, vomiting, and diarrhea—occur in approximately 20–30% of patients, are primarily mediated through delayed gastric emptying, and early treatment discontinuation remains common, underscoring the importance of individualized counseling and dose titration (79). Of particular concern, severe gastrointestinal adverse events may alter the time to peak concentration and peak concentration of tacrolimus, increasing fluctuations in blood drug levels and the attendant risk of rejection (85).

#### 5.2.3 Safety

With respect to safety, SGLT2 inhibitors warrant particular attention to the risks of genitourinary infections and volume depletion. A single-center retrospective study involving 130 kidney transplant recipients reported that 10% of patients developed urinary tract infections within 12 months of drug initiation, and 5.4% discontinued therapy due to recurrent urinary tract infections (73). Another comparative study of 240 kidney transplant recipients receiving SGLT2 inhibitors versus standard care found that although glycemic control was superior in the SGLT2 inhibitor group, volume depletion and genitourinary infections remained important clinical concerns (74). A systematic review and meta-analysis published in 2025 demonstrated that SGLT2 inhibitors were not associated with a significantly increased risk of urinary tract infection, genital mycotic infection, urosepsis, or graft rejection in kidney transplant recipients; however, the included studies were limited by small sample sizes and short follow-up durations (75). Currently, the KDIGO guidelines recommend SGLT2 inhibitors for patients with chronic kidney disease and diabetes, but these recommendations have not been extended to kidney transplant recipients, as this population was excluded from the pivotal clinical trials (77).

A study of 76 solid organ transplant recipients (74% kidney transplant recipients) demonstrated that when GLP-1 receptor agonists were co-administered with tacrolimus, the median tacrolimus trough concentration remained similar; within 12 months of GLP-1 receptor agonist initiation, four patients (5.3%) experienced rejection, and the 12-month patient and graft survival rates were 97.4% and 100%, respectively (85). The authors concluded that the combination of GLP-1 receptor agonists with immediate-release tacrolimus may be safe for diabetes management in solid organ transplant recipients, although larger studies are required for further validation (85). The current evidence for GLP-1 receptor agonists is also constrained by small sample sizes, short follow-up periods, and potential selection bias (79).

#### 5.2.4 Therapeutic implications and challenges

The incorporation of SGLT2 inhibitors and GLP-1 receptor agonists into the comprehensive management of kidney transplant recipients represents a paradigm shift from addressing rejection and metabolic disturbances separately toward an integrated immunometabolic regulatory strategy. From a mechanistic perspective, the inhibitory effects of both drug classes on multiple nodes—including NLRP3/NF-κB/complement/adhesion molecule pathways—may theoretically attenuate the metabolic stress-induced “priming” of the graft endothelium, thereby reducing the risk of DSA-mediated ABMR initiation or progression (i.e., mitigation of the “first hit” in the “two-hit” model).

However, translating these potential benefits into clinical evidence faces multiple challenges. First, randomized controlled trials designed with ABMR-related endpoints (e.g., microvascular inflammation scores, molecular ABMR scores, *de novo* DSA incidence, dd-cfDNA levels) as primary or key secondary outcomes are required; an exploratory study is currently evaluating the effects of SGLT2 inhibitors on graft inflammation and fibrosis markers in kidney transplant recipients (NCT04965935). Second, trial designs should employ stratified randomization according to recipients’ immunological risk (sensitization status, prior rejection history) and metabolic phenotype (presence or absence of PTDM/metabolic syndrome) to identify the populations most likely to derive benefit. Third, given the low incidence of ABMR events, the use of composite endpoints and extended follow-up periods (e.g., 3–5 years) is essential. Finally, should these agents prove effective in improving graft outcomes, their real-world implementation will require addressing concerns of transplant nephrologists regarding adverse effects, drug cost-effectiveness, and the development of standardized guideline recommendations. Future pragmatic clinical trials, driven by the central hypothesis that metabolic improvement slows ABMR progression, will be instrumental in defining the precise role of these agents within integrated immunometabolic strategies. In the TEDDY trial (NCT04965935), kidney transplant recipients are being randomized to dapagliflozin or placebo and followed for a planned duration of three years, with the primary endpoint being a composite assessment of eGFR slope, changes in proteinuria, and histological and molecular markers of graft inflammation and fibrosis; this trial will provide the first prospective evidence on the long-term renoprotective effects of SGLT2 inhibitors in kidney transplantation.

These data support the metabolic benefits and acceptable safety of SGLT2 inhibitors and GLP-1 receptor agonists in kidney transplant recipients, though careful monitoring of volume status, urinary tract infection risk, and gastrointestinal tolerability remains warranted.It is important to note that the current evidence is constrained by small sample sizes, short follow-up periods, and potential selection bias, all of which reduce the certainty of safety and efficacy assessments (79).

#### 5.2.5 Others

For dyslipidemia, beyond statins and ezetimibe, PCSK9 inhibitors (like evolocumab) are a cutting-edge therapy to drastically lower LDL cholesterol. Case reports and small series in kidney transplant patients indicate that PCSK9 inhibitors achieve significant lipid reduction without notable adverse interactions with immunosuppressants (38). A network meta-analysis published in 2025, encompassing 16 randomized controlled trials with a total of 79,615 patients, demonstrated that PCSK9 inhibitors significantly reduce LDL-cholesterol in solid organ transplant recipients, with evolocumab ranking highest for both LDL-C reduction (surface under the cumulative ranking curve [SUCRA] 67.2%) and reduction in cardiovascular events (SUCRA 69.5%), without an increased risk of acute rejection or infection (80). These data affirm the efficacy and safety of PCSK9 inhibitors in the transplant population, offering an important therapeutic alternative for patients with refractory hypercholesterolemia or statin intolerance. It must be recognized, however, that studies of PCSK9 inhibitors in kidney transplant recipients are largely confined to case reports and small-scale investigations, and robust evidence from large randomized controlled trials is lacking (81). Moreover, patients with severe chronic kidney disease and kidney transplant recipients were excluded from the major PCSK9 inhibitor clinical trials, further limiting the strength of the available evidence (82). Thus, while existing data support the potential utility of PCSK9 inhibitors in the transplant population, their use should be reserved for patients with statin intolerance or refractory hypercholesterolemia pending the availability of more definitive safety data. These agents may be particularly useful in patients with refractory hypercholesterolemia or those intolerant of high-dose statins. Finally, aggressive management of obesity and weight gain is being examined. Supervised exercise programs and dietary counseling tailored to transplant recipients can curtail excessive weight gain. In a few cases of morbid obesity, bariatric surgery after transplant has been explored as a means of improving metabolic profiles, but with meticulous management of surgical risk and immunosuppression adjustment (39).

### 5.3 Optimizing immunosuppression

Another key strategy is refining immunosuppressive regimens to balance efficacy and metabolic side effects. As mentioned, the use of belatacept (a costimulation blockade agent) in place of a calcineurin inhibitor is one approach to avoid CNI-induced metabolic complications; trials have shown that belatacept-based regimens result in better metabolic profiles (lower incidence of PTDM and dyslipidemia) at the cost of a slightly higher early rejection risk (56). Similarly, steroid-minimization protocols (early steroid withdrawal or steroid-free maintenance) are employed to reduce long-term metabolic harm, although they must be limited to low-immunologic-risk patients to be safe. Ongoing research is evaluating novel immunosuppressants and cell therapies that could induce tolerance or specifically prevent ABMR without the broad metabolic toxicities of current drugs (11). The ultimate goal is an immunosuppressive regimen that safeguards the graft against rejection and also maintains the metabolic well-being of the patient.

Long-term multidisciplinary care and research is required. Transplant doctors, cardiologists, and endocrinologists must work together to screen and treat cardiovascular and metabolic risk factors in transplant recipients, in the same way that they work together to prevent rejection (3). Emerging therapies, both immunological and metabolic, offer hope that the historic trade-offs between preventing rejection and causing metabolic disease can be mitigated. Clinical trials focused on transplant-specific use of these therapies (such as SGLT2 inhibitors or complement inhibitors) will be critical to establish best practices (33). In the meantime, a proactive approach to both immunologic and metabolic health after KT stands as the best strategy to enhance graft longevity and patient survival.

## 6 Challenges and research gaps

Despite the advancement of knowledge and treatment of both ABMR and metabolic complications, much remains problematic. Among the major issues is that there is no specific approved therapy for chronic ABMR (6, 11). The majority of the interventions (from complement inhibitors to cell therapies) remain investigational, and clinical trials to convincingly improve long-term graft survival in patients with chronic active ABMR have largely given unencouraging results (11). Thus, treating physicians are often forced to use off-label and combination therapy in refractory rejection, with variable success (21). There is a great need for new drugs that inhibit or modulate the humoral immune response responsible for ABMR more effectively with less associated toxicity (13, 56).

Another difficulty is balancing the intensity of immunosuppression against metabolic side effects. Excessive immunosuppression immunologically protects the graft but can cause severe diabetes, hyperlipidemia, or other comorbidities; less immunosuppression prevents some of these metabolic complications but at the risk of rejection (56). Different patients have different immunologic risk and metabolic susceptibility, such that a single regimen fits none optimally. Research is needed to personalize immunosuppressive protocols – for example, using biomarkers to identify patients who could safely minimize steroids or calcineurin inhibitors without raising rejection risk (24, 56). Likewise, more data are needed on whether aggressive control of metabolic factors (like tight glycemic control or weight loss interventions) can directly improve graft outcomes. Currently, much of the rationale for managing metabolic risk in transplant patients is extrapolated from cardiovascular benefits in the general population, rather than proven reductions in rejection or graft loss specifically (38, 71).

In addition, longer-term investigations will be required to further define the impact of newer interventions on both domains. For instance, while SGLT2 inhibitors and GLP-1 receptor agonists appear promising in PTDM, their long-term effects on graft function and immune markers in transplant recipients remain under investigation (33, 71). Similarly, novel anti-rejection regimens (e.g., costimulatory pathway antagonists or complement cascade inhibitors) need to be investigated not only for their immunologic efficacy but also for their metabolic side-effect profiles. Care integration is another area for improvement: transplant centers must coordinate care for comorbidities, but in practice, metabolic issues may be undermanaged due to an overwhelming focus on immunological control (3). Guidelines explicitly tailored to transplant recipients for management of diabetes, hypertension, and dyslipidemia are somewhat limited, and clinical trial enrollment of transplant patients in studies of cardiometabolic therapies has been low (39). Encouragingly, some recent trials have started to include transplant-specific cohorts, which will help generate evidence-based recommendations (38).

In summary, key research areas that are deficient include: (1) the identification of efficacious therapies in chronic ABMR; (2) the optimal approach to individualizing immunosuppression based on patient risk status; (3) determination of whether improvement of metabolic health can lead to a decrease in rejection or loss of the graft; and (4) long-term efficacy and safety information regarding use of new metabolic and immunologic treatments among transplant recipients. These will require collaborative studies and multicenter trials to fill. Until then, clinicians must navigate these challenges using the best available evidence and a multidisciplinary, case-by-case approach.

Notably, an important and long-standing unresolved question persists: why, in contemporary transplant practice, is there still a profound paucity of large-scale, head-to-head randomized controlled trials comparing metabolically favorable immunosuppressive regimens—for instance, belatacept versus tacrolimus, or the novel agent tegoprubart versus tacrolimus? The factors contributing to this research gap are multifaceted. First, kidney transplant recipients have been systematically excluded from pivotal clinical trials—for example, the major cardiovascular outcome trials of SGLT2 inhibitors and GLP-1 receptor agonists, as well as the principal trials of PCSK9 inhibitors, all excluded transplant recipients (77, 82). Second, industry-sponsored head-to-head trials are predominantly focused on active-comparator studies required for regulatory approval, and metabolic outcomes are typically incorporated only as secondary endpoints or safety measures, with a conspicuous absence of trials specifically designed with metabolic improvement as the primary outcome. Third, the heterogeneity of the transplant population—encompassing variations in donor type, immunological risk stratification, and comorbidity burden—increases the complexity of trial design and necessitates larger sample sizes and longer follow-up periods to achieve adequate statistical power. Furthermore, regulatory agencies have yet to define the weight that metabolic endpoints should carry in the evaluation of immunosuppressive regimens for transplantation.

In recent years, this landscape has begun to shift. The BESTOW trial (NCT05983770), a phase 2 head-to-head study of tegoprubart versus tacrolimus in kidney transplantation involving 127 recipients, demonstrated that the incidence of new-onset diabetes was significantly lower in the tegoprubart group compared with the tacrolimus group (2.1% vs. 16.7%), with correspondingly lower rates of hypertension, tremor, and cardiovascular toxicity. Similarly, head-to-head comparisons of belatacept versus tacrolimus have shown that belatacept-based regimens confer superior preservation of renal function (median estimated glomerular filtration rate at 36 months: 66 vs. 53 mL/min/1.73 m²). Nevertheless, in these studies, metabolic endpoints have largely remained secondary outcomes, and follow-up durations have been limited. Future research should prioritize large-scale clinical trials that adopt metabolic improvement as the primary endpoint and employ pragmatic designs—for instance, leveraging registry-based data or electronic health records—to definitively establish the long-term benefits of metabolically favorable immunosuppressive regimens in real-world settings.

## 7 Conclusion

Metabolic dysregulation and ABMR are dual threats to the longevity of kidney allografts. These processes, once thought of separately, are increasingly recognized as interrelated. ABMR remains a formidable clinical problem, particularly in the chronic form, and is a major cause of late graft failure. Meanwhile, post-transplant diabetes mellitus (PTDM) is a frequent complication that contributes substantially to recipient morbidity and, potentially, to allograft injury. Care in one arena alone is not sufficient; optimal post-transplant care is utilizing an integrative approach to both immunologic and metabolic health.

Ongoing research and development are gradually but relentlessly arming clinicians with more potent tools with which to meet these challenges. Novel immunotherapeutics (complement inhibitors, antibody-depleting enzymes, cytokine blockers, etc.) and improved diagnostic assays hold the promise of improved control of ABMR. Meanwhile, newer cardiometabolic therapies and lifestyle interventions have the potential to improve post-transplant metabolic syndrome and its constituents. The last step will be to integrate these advances into individualized care plans that maximize graft survival and cardiovascular risk minimization.

In short, maximizing kidney transplant outcome requires attention on two fronts: preventing and treating alloimmune injury, and maintaining metabolic well-being. By understanding their interplay and taking advantage of novel treatment in both areas, the transplant community can move closer to the long-term ideal of KT—maintaining graft function and enhancing patient survival far into the future beyond current capabilities.

### 7.1 Search strategy and literature selection

For this narrative review, literature searches were conducted in PubMed, Web of Science, and Embase for articles published between January 2010 and April 2026. Search terms included “kidney transplantation,” “antibody-mediated rejection,” “metabolic syndrome,” “post-transplant diabetes mellitus,” “dyslipidemia,” “hypertension,” “gut microbiome,” “complement activation,” “SGLT2 inhibitor,” “GLP-1 receptor agonist,” “PCSK9 inhibitor,” and their combinations. Priority was given to randomized controlled trials, systematic reviews and meta-analyses, major professional society guidelines (e.g., Banff meeting reports), and landmark prospective cohort studies published in peer-reviewed journals. Select foundational mechanistic studies (e.g., on the role of AGEs in chronic renal transplant dysfunction and CD55/CD59 in complement regulation) were also included because they provide essential conceptual underpinnings. Case reports were cited only when they offered unique clinical insights into specific adverse events or emerging therapies (e.g., early experience with PCSK9 inhibitors in kidney transplant recipients). Two authors independently screened titles and abstracts, and disagreements were resolved by consensus or by a third author. Consistent with the narrative nature of this review, formal risk-of-bias assessment and quantitative synthesis were not performed.
